# *In situ* high-temperature EXAFS measurements on radioactive and air-sensitive molten salt materials

**DOI:** 10.1107/S160057751801648X

**Published:** 2019-01-01

**Authors:** Anna L. Smith, Malte N. Verleg, John Vlieland, Dick de Haas, Jaen A. Ocadiz-Flores, Philippe Martin, Jörg Rothe, Kathy Dardenne, Mathieu Salanne, Aimen E. Gheribi, Elisa Capelli, Lambert van Eijck, Rudy J. M. Konings

**Affiliations:** a Delft University of Technology, Faculty of Applied Sciences, Radiation Science and Technology Department, Mekelweg 15, 2629 JB Delft, The Netherlands; b CEA, Nuclear Energy Division, Research Department on Mining and Fuel Recycling Processes, SFMA, LCC, F-30207 Bagnols-sur-Céze, France; c Karlsruhe Institute of Technology (KIT), Institute for Nuclear Waste Disposal (INE), Radionuclide Speciation Department, Hermann-von-Helmholtz-Platz 1, 76344 Eggenstein-Leopoldshafen, Germany; d UPMC Université Paris 06, CNRS, ESPCI, UMR 7195, PECSA, 75005 Paris, France; e Centre for Research in Computational Thermochemistry, Department of Chemical Engineering, Ecole Polytechnique, CP 6079, Succursale ‘Downtown’, Montreal (Quebec), Canada H3C 3A7; f European Commission, Joint Research Centre Karlsruhe (JRC), PO Box 2340, D-76125 Karlsruhe, Germany

**Keywords:** XAFS, fluoride salts, molten salt reactor, neutron diffraction, molecular dynamics

## Abstract

The development at the Delft University of Technology (TU Delft, The Netherlands) of an experimental set-up dedicated to high-temperature *in situ* EXAFS measurements of radioactive, air-sensitive and corrosive fluoride salts is reported. A detailed description of the sample containment cell, of the furnace design, and of the measurement geometry allowing simultaneous transmission and fluorescence measurements is given herein. The performance of the equipment is tested with the room-temperature measurement of thorium tetrafluoride, and the Th—F and Th—Th bond distances obtained by fitting of the EXAFS data are compared with the ones extracted from a refinement of neutron diffraction data collected at the PEARL beamline at TU Delft. The adequacy of the sample confinement is checked with a mapping of the thorium concentration profile of molten salt material. Finally, a few selected salt mixtures (LiF:ThF_4_) = (0.9:0.1), (0.75:0.25), (0.5:0.5) and (NaF:ThF_4_) = (0.67:0.33), (0.5:0.5) are measured in the molten state. Qualitative trends along the series are discussed, and the experimental data for the (LiF:ThF_4_) = (0.5:0.5) composition are compared with the EXAFS spectrum generated from molecular dynamics simulations.

## Abstract 2

An experimental set-up and specific sample containment allowing high-temperature *in situ* EXAFS measurements of radioactive, air-sensitive and corrosive fluoride salts is described. First results are reported and compared with molecular dynamics simulations of the highly disordered liquid salts.

## 1. Introduction   

Faced with the huge energy challenge of our century and the need for increased safety margins, the nuclear community is currently working on the development of a new generation of nuclear reactors, the so-called Generation IV reactor systems, that should replace the current fleet of reactors at the end of their operating licences. The Generation IV International Forum (GIF) defined four main criteria for these new systems in the areas of sustainability, economics, safety and reliability, and proliferation resistance, and selected six designs that satisfied their requirements (GIF, 2013 ▸, 2014 ▸). Among these, the molten salt reactor (MSR) is a highly innovative concept (Renault *et al.*, 2009 ▸), that uses a fluoride or chloride molten salt as opposed to the usual ceramic (U,Pu)O_2_ fuel considered for most of the Generation IV concepts. Operated at a temperature around 550–800°C, this salt serves both as fuel and primary coolant, and shows a number of features that make the design inherently safer: low vapour pressure, strong negative temperature coefficient and possibility to drain the liquid fuel into emergency dump tanks ensuring that the reactor remains subcritical in an accidental event or temperature transient, possibility to perform online fuel cleanup by separating the fission products, hence avoiding excessive heat generation by radioactive decay (Beneš & Konings, 2012 ▸; Dolan, 2017 ▸). In addition, the MSR can run on a thorium fuel cycle, which leads to much less long-lived radioactive waste production (Delpech *et al.*, 2009 ▸), and ensures a sustainable energy production (thorium concentrations on Earth being three times larger than uranium; NEA, 2015 ▸).

The choice of the fuel composition is critical for the performance of the reactor, and it must satisfy a number of requirements: advantageous neutronic properties, sufficiently low melting temperature, low vapour pressure, high heat capacity and thermal conductivity, adequate viscosity, sufficient solubility for fissile material (^235^U, ^233^U or ^239^Pu) (Beneš & Konings, 2012 ▸). The reference fuel retained in Europe for the molten salt fast reactor design includes enriched lithium (^7^Li isotope), uranium, thorium and plutonium fluoride ^7^LiF–UF_4_–ThF_4_–(PuF_3_) (Delpech *et al.*, 2009 ▸), but a wide variety of other compositions are currently under investigation worldwide (Dolan, 2017 ▸). One main challenge for the development of this technology and its commercialization in the near future is a thorough understanding of the liquid fuel physico-chemical properties. In particular, only a limited number of experimental and theoretical studies have been reported in the literature on the structure of the molten salt, mainly because of the challenges associated with the handling of such material (radioactive, hygroscopic and highly corrosive at high temperature), which requires a dedicated infrastructure.

These molten salts constitute essentially ionic liquids in which cations and anions form a loose network (Beneš & Konings, 2012 ▸). Depending on conditions of temperature and concentration, they can form a dissociated ionic melt, molecular species or exhibit polymerization (Salanne *et al.*, 2007 ▸; Dracopoulos *et al.*, 1998 ▸), which is directly linked to their viscosity, a crucial property for the design of the reactor. For instance, molecular dynamics (MD) studies on the LiF–BeF_2_ system by Salanne *et al.* (2007 ▸) have shown that at low BeF_2_ content the salt is mainly composed of Li^+^, BeF_4_^2−^ and F^−^ species, while polymerization was occurring with increasing BeF_2_ concentration. Be_2_F_7_^3−^, Be_3_F_10_^7−^, Be_4_F_13_^5−^ units form at higher BeF_2_ content, leading to a high viscosity of the corresponding salt (Beneš & Konings, 2012 ▸).

In this work, we report the development of a dedicated experimental set-up to investigate the local structure of radioactive fluoride salts at high temperatures using extended X-ray absorption fine-structure (EXAFS) spectroscopy. EXAFS is an element-specific technique recording electronic transitions. It probes the order at short and intermediate ranges, and is particularly well adapted to determine coordination number, nature of neighbouring atoms, disorder (Bunker, 2010 ▸), and to identify the formation of molecular complexes in the melt (Rollet *et al.*, 2005 ▸). A furnace set-up with a specific geometry was designed at the Delft University of Technology (TU Delft, The Netherlands) to accommodate the safety rules, the constraints imposed by the investigated materials and the measurements conditions at the INE beamline (Rothe *et al.*, 2012 ▸) at the Karlsruhe Research Accelerator (KARA) [Karlsruhe Institute of Technology (KIT), Germany]. Nevertheless, this set-up, which follows the rule of two independent confinement barriers, could be very easily used at other beamlines dedicated to radioactive materials, such as the MARS beamline at the French National Synchrotron Facility SOLEIL (Paris, France), the BL27B beamline at the Photon Factory, KEK (Tsukuba, Japan), and the ROBL-BM20 beamline at the European Synchrotron Research Facility (Grenoble, France).

A wide variety of furnace and cell designs can be found in the literature for *in situ* XAFS measurements on solids and liquids under vacuum, inert gas atmosphere, air or controlled oxygen potential (Berry *et al.*, 2003 ▸). Only a limited number of set-ups have been developed, however, for the measurement of halide salts at high temperatures. Studies of chloride and bromide salts have been performed using quartz cells (Okamoto *et al.*, 2000 ▸, 2001 ▸; Ablanov *et al.*, 1999 ▸) or a pellet of boron nitride containing the halide salt dispersed and confined inside the porous network of the BN matrix (Cicco *et al.*, 1996 ▸, 1997 ▸). The former option is not suitable for the corrosive fluoride salts, however. Molten metal fluorides have therefore been measured embedded inside a BN matrix (Watanabe *et al.*, 2006 ▸; Matsuura *et al.*, 2009 ▸). An additional containment becomes necessary to preserve the integrity of the investigated material, however, as fluoride salts are also highly hygroscopic, meaning that they are prone to oxidation reaction and react with moisture. Bessada and co-workers have used an airtight cell surrounding the BN pellet, consisting of two plates of boron nitride fixed hermetically with stainless steel screws (Rollet *et al.*, 2004 ▸; Bessada *et al.*, 2017 ▸). Finally, the measurement of radioactive materials such as thorium or uranium fluorides imposes additional constraints and the inclusion of multiple-containment barriers into the design. A very limited number of EXAFS measurements have been reported in the literature of molten radioactive fluoride salts (Numakura *et al.*, 2011 ▸, 2014 ▸; Matsuura *et al.*, 2013 ▸; Bessada *et al.*, 2017 ▸). They have all been performed in transmission, on molten salt material confined inside the BN matrix of a pellet, itself contained in the aforementioned air-tight BN cell.

In this work, we report for the first time measurements on pure materials as opposed to confined inside a BN matrix, and a design allowing simultaneous transmission and fluorescence EXAFS measurements. A detailed description of the furnace and sample containment cell is firstly given hereafter. Second, the performance of the set-up is tested with the measurement of thorium tetrafluoride at room temperature. The adequacy of the sample confinement is checked with a mapping at room temperature of the thorium concentration profile of two melted (NaF:ThF_4_) salt mixtures. Finally, the EXAFS data of selected molten compositions (LiF–ThF_4_) = (0.9:0.1), (0.75:0.25), (0.5:0.5) and (NaF:ThF_4_) = (0.67:0.33), (0.5:0.5) are presented, and the results of the (LiF:ThF_4_) = (0.5:0.5) composition are compared with the spectrum generated by molecular dynamics simulations.

## 2. Experimental method   

A dedicated experimental set-up was designed in this work to accommodate the specific requirements of high-temperature XAS measurements on radioactive molten salt materials. Radioprotection rules impose the use of three safety barriers for the radioactive material which in our design are: a boron nitride cell containing the sample for the first containment (see Section 2.2), the furnace for the second containment (see Section 2.1), and a surrounding glovebox (see Section 2.4) for the third containment.

### 2.1. Furnace design   

**Figure 1:**
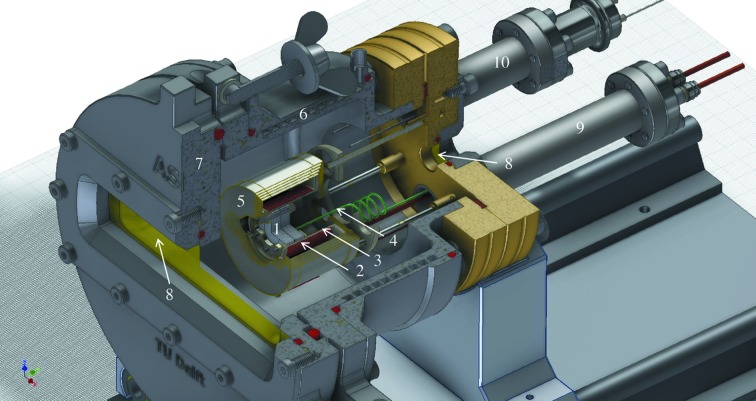
Furnace design for *in situ* high-temperature EXAFS measurements: (1) boron nitride cell, (2) Pt/Rh heating coil, (3) alumina tube, (4) Pt/Rh thermocouple, (5) molybdenum heating shields, (6) water cooling, (7) stainless steel casing, (8) Kapton windows (75 µm), (9) copper feedthrough, (10) thermocouple feedthrough.

The furnace design, developed at the Delft University of Technology (TU Delft, The Netherlands), is shown in Fig. 1 ▸. The sample placed in a boron nitride measurement cell (see Section 2.2) is heated under high vacuum inside an alumina tube (22 mm inner diameter, 24.75 mm outer diameter and 28.5 mm length) surrounded by a 0.8 mm Pt/Rh (10%) heating coil comprising nine windings. The extremities of the wire are clamped to copper feedthroughs at the back of the furnace. The vacuum inside the furnace chamber (∼2 × 10^−5^ mbar) ensures that the highly hygroscopic fluoride salts retain their purity during the measurements, and do not oxidize or form oxyfluorides at high temperatures. The alumina tube is surrounded by five cylindrical thermal shields made of molybdenum, and the stainless steel walls of the furnace are cooled with water. Kapton windows with thickness 75 µm, which are transparent to X-rays [the attenuation length at 16.3 keV is equal to 8.56 mm (Ravel & Newville, 2005 ▸)] and can resist temperatures up to ∼250°C, are used at the front and back of the furnace to let the X-rays pass through from the first to the second ionization chamber. The rectangular entry window (with dimensions 124 mm × 24 mm) and the circular exit window (with diameter 11 mm) are sealed with Teflon (PTFE) O-rings. Five additional heating shields are moreover placed at the rectangular entry window, with rectangular openings ranging from 48 mm × 4 mm to 75 mm × 3 mm. The temperature is measured with a R-type Pt/Rh (13%) thermocouple placed in contact with the boron nitride cell. The temperature is controlled using a power supply: the voltage is set through the heating wire, and the current is regulating itself to the changing resistance of the wire. The temperature reading was checked by measuring the melting points of standard materials (Au, Ag, In, Al). The uncertainty on the temperature is estimated to be around ±10°C. The temperature stability for a measurement time of around 4 h is estimated to be around ±5°C at 1100°C.

### 2.2. Sample containment cell   

**Figure 2:**
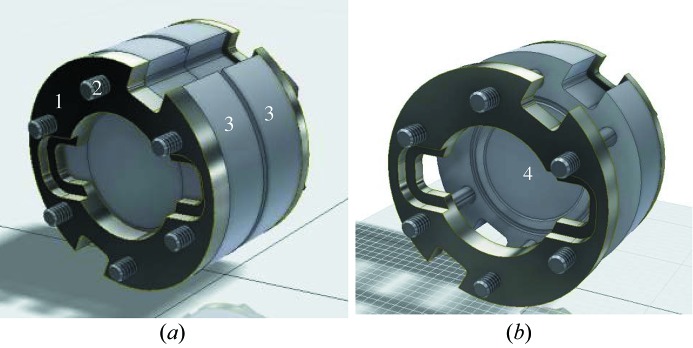
Sample containment cell: (1) molybdenum ring, (2) molybdenum bolts, (3) half-cell in boron nitride, (4) emplacement for sample pellet of 100 µm thickness.

A dedicated containment cell has also been developed (Fig. 2 ▸) that meets several criteria. The cell serves as first barrier for the fluoride thorium-containing salts, a radioactive and particularly hygroscopic material, and must therefore be completely air tight and leak tight at high temperatures. The material chosen, *i.e.* boron nitride, is known to be compatible with the corrosive fluoride salts (Beneš *et al.*, 2010 ▸), shows a high thermal conductivity, and absorbs little X-rays at the ranges of energies of interest in this work [the attenuation length at 16.3 keV is equal to 5.87 mm (Ravel & Newville, 2005 ▸)]. A high grade of boron nitride without binder (HeBoSint BN D100; Henze Boron Nitride Products) was chosen to avoid chemical reaction with the salt or spurious absorption of the X-ray beam. The containment geometry consists of two boron nitride plates of thickness 5 mm, one with a flat surface, the other with a 100 µm deep circular reservoir of diameter 10 mm, where the sample is placed. An additional groove (outside diameter 12.8 mm) is also present outside this reservoir, that can contain excess salt if necessary, hence avoiding the risk of a possible leak of the liquid salt. The two half-cells are fixed together with molybdenum rings and bolts that can withstand the high temperatures (600°C < *T* < 1200°C) of the measurements.

### 2.3. Sample preparation   

The samples were prepared using commercial lithium fluoride (LiF, Alfa Aesar, 99.99% metals basis, ultra dry), sodium fluoride (NaF, Alfa Aesar, 99.99%, metals basis) and thorium fluoride (ThF_4_, International Bio-Analytical Industries Inc., 99.99%) materials. The lithium fluoride was used as delivered, while the sodium fluoride was subjected to a pretreatment at 400°C for 4 h in an open nickel boat under argon atmosphere to remove residual moisture. The thorium fluoride, which shows a tendency to oxidize or form oxyfluorides easily, was moreover purified using NH_4_HF_2_ as fluorinating agent, as described in detail by Capelli *et al.* (2013 ▸). Because of the high sensitivity of the fluoride salts to water and oxygen, they were handled exclusively in the dry atmosphere of an argon-filled glove box, where water and oxygen content are kept below 1 p.p.m. The samples’ purities were checked using powder X-ray diffraction (XRD), neutron diffraction (only for ThF_4_) and differential scanning calorimetry (DSC) measurements. No secondary phases were detected by XRD, while the DSC data showed single events with melting temperatures of (845 ± 5)°C (LiF), (995 ± 5)°C (NaF) and (1107 ± 5)°C (ThF_4_), in very good agreement with the literature data [848.3°C for LiF (Chase, 1998 ▸), 996°C for NaF (Chase, 1998 ▸), (1110 ± 3)°C for ThF_4_ (Konings *et al.*, 2005 ▸)].

Several intermediate compositions, (LiF–ThF_4_) = (0.9:0.1), (0.75:0.25), (0.5:0.5) and (NaF–ThF_4_) = (0.67:0.33), (0.5:0.5), were prepared by mixing the end-members in the appropriate ratios. The mixtures were subsequently pressed (at 8 tons cm^−2^) into pellets (8–20 mg) of thickness less than 100 µm and placed inside the reservoir of the BN containment cell. In contrast to the encapsulation methods reported in the literature for *in situ* EXAFS measurements of zirconium and thorium fluoride salts (Pauvert *et al.*, 2011 ▸; Numakura *et al.*, 2011 ▸; Bessada *et al.*, 2017 ▸), the samples were not mixed with boron nitride powder but measured as pure salts, which ensures a more homogeneous distribution upon melting.

### 2.4. Measurement configuration: simultaneous transmission and fluorescence measurements   

**Figure 3:**
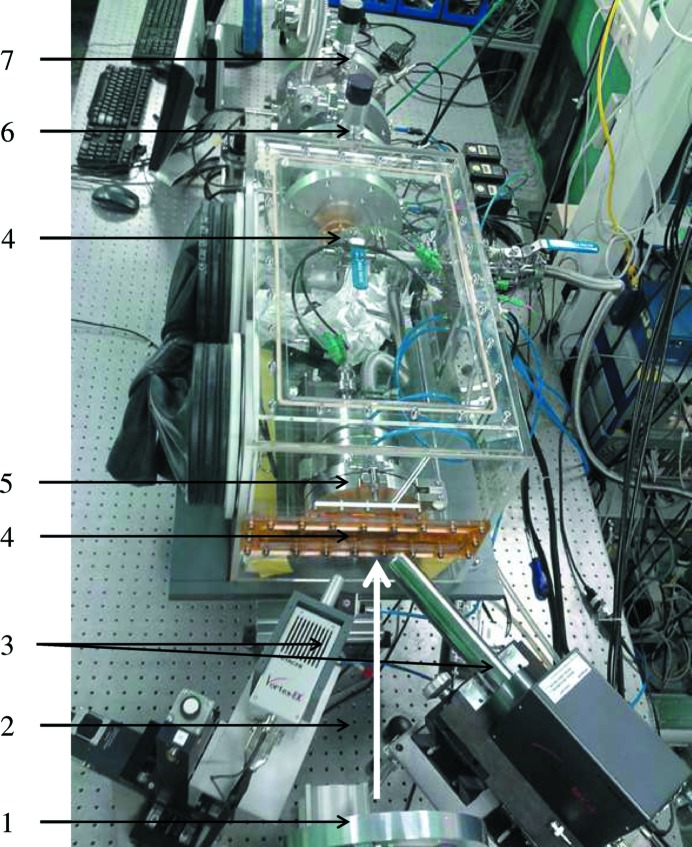
Measurement configuration at the INE beamline: (1) first ionization chamber, (2) impinging X-ray beam, (3) fluorescence detectors, (4) Kapton windows of the glove box, (5) furnace, (6) second ionization chamber, (7) third ionization chamber.

The experimental set-up developed in this work allows the simultaneous measurement of the transmission and fluorescence signals thanks to the two Kapton windows in the front and back of the furnace (Fig. 1 ▸) and glovebox (Fig. 3 ▸). Similar designs reported in the literature for measurements of zirconium- or thorium-containing salts using a tubular furnace were limited to measurement configurations in transmission only (Pauvert *et al.*, 2011 ▸; Numakura *et al.*, 2011 ▸; Bessada *et al.*, 2017 ▸). The incident and transmitted X-ray beams are detected by two ionization chambers filled with Ar. The fluorescence silicon drift detectors (one four-element and one single SDD vortex) are placed next to the first ionization chamber and facing the front Kapton window of the furnace at a distance around 20 cm. This special configuration allows a good quality signal to be obtained for a variety of sample compositions, from concentrated materials for which transmission measurements are preferred, to very diluted materials for which fluorescence detection is more adapted.

**Figure 4:**
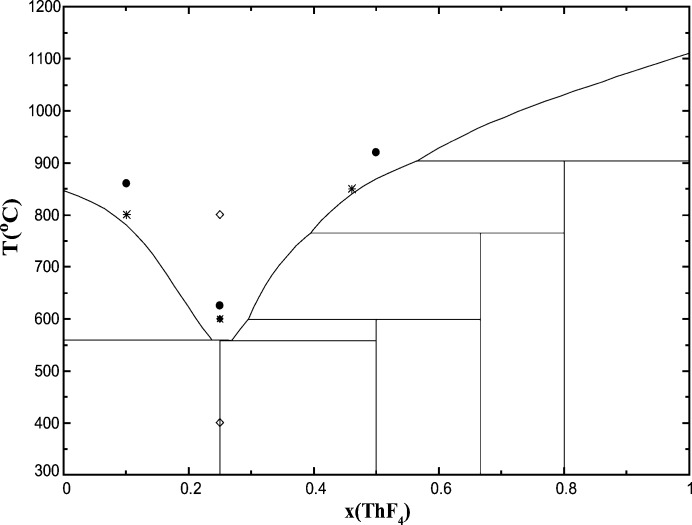
LiF–ThF_4_ phase diagram as optimized in the work of Capelli *et al.* (2014 ▸). Filled circles: compositions measured in this work; open diamonds: compositions reported by Matsuura *et al.* (2013 ▸); stars: compositions reported by Numakura *et al.* (2014 ▸): note that the EXAFS data are not shown in this reference, and the temperature of the measurement is not specified.

Measurements were performed at the INE beamline (Rothe *et al.*, 2012 ▸) at KARA, Germany. The storage ring operating conditions at KARA were 2.5 GeV and 150–170 mA. The INE beamline (Rothe *et al.*, 2012 ▸) is equipped with a Ge(422) double-crystal monochromator (DCM) coupled with a collimating and a focusing Rh-coated mirrors before and after the DCM, respectively. The DCM crystals were detuned at 70% of the maximum intensity of the rocking curve, and the incident beam intensity was held constant by means of a piezo-driven feedback system to the second crystal. The beam spot size was 300 µm × 500 µm. The XAS spectra were collected at the Th *L*_3_-edge (16.3 keV), in the energy range 16.1 to ∼17.05 keV, at high temperatures, *i.e.* 50–100°C above the melting point of the investigated composition (Figs. 4 ▸ and 5 ▸), and after cooling back down to room temperature. After reaching the measurement temperature, an equilibration time of about 15 min was employed before collecting the XAS data, so as to ensure proper homogenization of the sample. A step size of 0.8 eV was used in the XANES region. The energy *E*_0_ of the edge absorption threshold position was taken at the first inflection point of the spectrum by using the first node of the second derivative. Several acquisitions (three to four spectra in the liquid, two to three spectra in the solid) of about 1 h each were collected on the same sample and summed up to improve the signal-to-noise ratio. Before averaging the scans, each spectrum was aligned using the XANES spectrum of a thorium dioxide pellet (ThO_2_ diluted in boron nitride powder) located between the second and third ionization chambers, and measured at the same time as the sample. The *ATHENA* software (Version 0.9.22) (Ravel & Newville, 2005 ▸) was used to normalize the spectra and extract the EXAFS data.

EXAFS data were collected in this work up to ∼12.5 Å^−1^, and were Fourier transformed using the Hanning window over the *k*-range 3–12.2 Å^−1^ (*dk* = 2) for solids, and 3–9 Å^−1^ (*dk* = 2) for the liquid salts given the limited short range order in the liquid state. To assess the quality of the collected data, curve fitting was performed on a reference ThF_4_ sample at room temperature and on the (LiF:ThF_4_) = (0.5:0.5) composition at 920°C, based on the standard EXAFS equation using the *ARTEMIS* software (Ravel & Newville, 2005 ▸) in *k* ^3^ space. Phases and amplitudes for the interatomic scattering paths were calculated with the *ab initio* code *FEFF8.40*. The shift in the threshold energy (Δ*E*_0_) was varied as a global parameter and the coordination numbers were optimized. The amplitude factor *S*_0_^2^ was fixed for all paths to 0.9. A single value of Δ*E*_0_ for all diffusion paths was allowed to vary. The Debye–Waller parameters σ^2^ were allowed to vary for each shell.

## 3. Molecular dynamics simulations   

MD simulations were performed for the (LiF:ThF_4_ = 0.5:0.5) composition at the corresponding experimental temperature, *i.e.* ∼50°C above the liquidus. The potential, derived from the polarizable ion model (PIM), consisted of a combination of charge–charge, dispersion, overlap repulsion and polarization, with the same functional forms and parameters as given by Dewan *et al.* (2013 ▸). Such parameters were validated by the authors by comparing calculated values for density, electrical conductivity, viscosity and heat capacity in the molten state in the LiF–ThF_4_ system with the available experimental data, with good results. The system was equilibrated for 500 ps in the NPT ensemble at 0 GPa and 920°C, from which the equilibrium volume was taken. This was followed by a 500 ps production run in the NVT ensemble at 920°C. Time steps in both ensembles were set to 0.5 fs, whereas the relaxation time for both the Nosé–Hoover thermostat and barostat (for the NPT run) was set to 10 ps. The cubic simulation cell contained 570 F^−^, 114 Th^4+^ and 114 Li^+^ ions in periodic boundary conditions. Cutoffs for the real space part of the Ewald sum and short-range potential were both set to half the length of the cell.

## 4. Performance and first results   

### 4.1. Room-temperature EXAFS measurement of thorium tetrafluoride   

**Figure 6:**
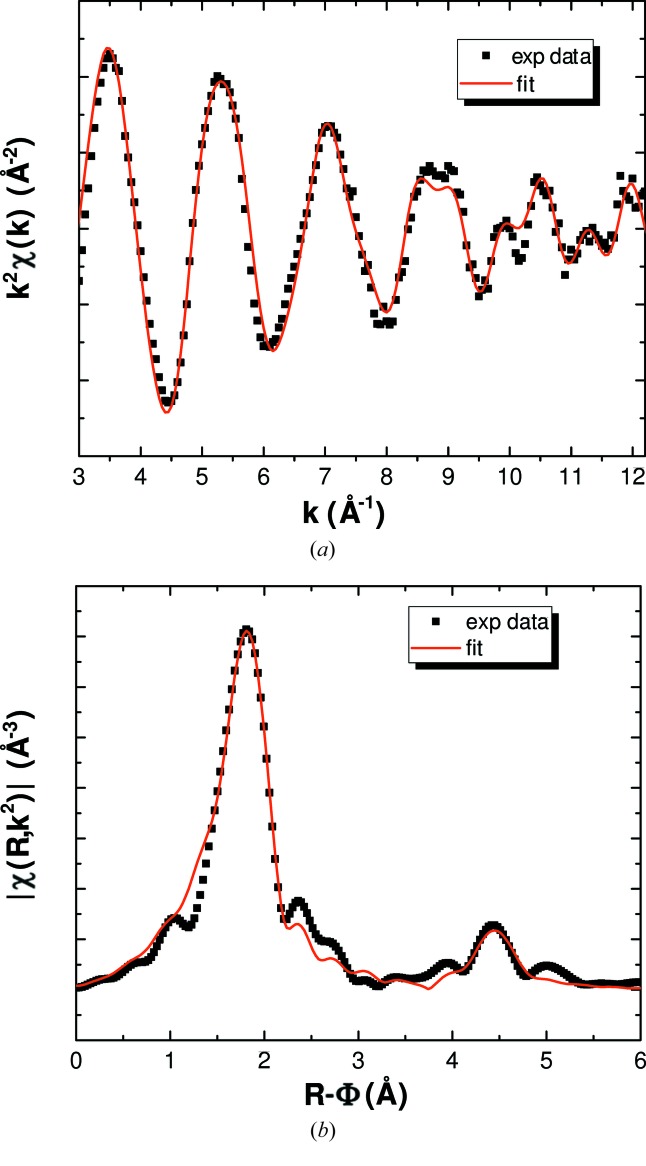
(*a*) Experimental and fitted *k* ^2^χ(*k*) spectra and (*b*) Fourier transform modulus of ThF_4_ at room temperature measured in transmission.

The performance of the developed experimental set-up was first tested with the room-temperature measurement of thorium tetrafluoride encapsulated in the BN cell, and placed inside the furnace chamber under vacuum. The collected transmission and fluorescence signals are shown in Fig. 15 in Appendix *A* [App appa]. The only difference between those signals is the slightly less intense amplitude for fluorescence due to self-absorption effects. Such results confirmed the suitability of the measurement configuration both for concentrated and diluted samples. The transmission data were subsequently fitted using the structural model described in the literature (Benner & Müller, 1990 ▸). The experimental and fitted *k* ^2^-weighted EXAFS spectrum and its Fourier transform (FT) are shown in Figs. 6(*a*) and 6(*b*) ▸.

**Figure 7:**
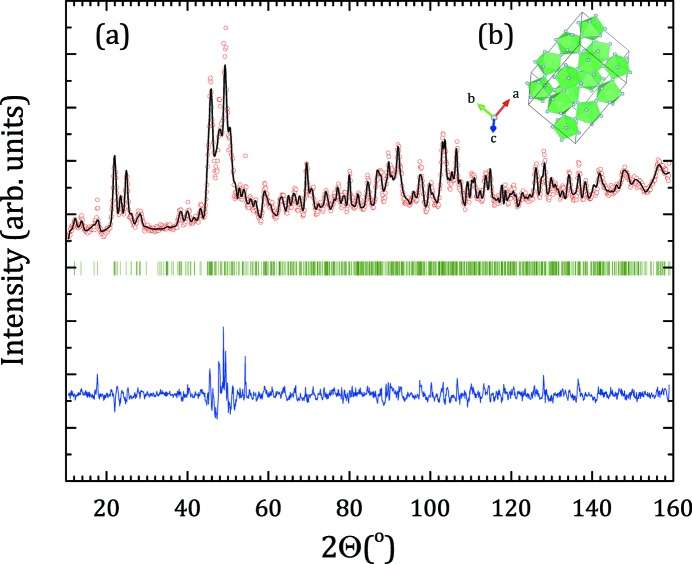
(*a*) Comparison between the observed (*Y*_obs_, in red) and calculated (*Y*_calc_, in black) neutron diffraction pattern of ThF_4_. *Y*_obs_ − *Y*_calc_, in blue, is the difference between the experimental and calculated intensities. The Bragg reflections’ angular positions are marked in green. Measurement at λ = 1.667 Å. (*b*) Sketch of the ThF_4_ structure showing the ThF_8_ polyhedra connected by their vertices in green and fluorine atoms in grey.

Thorium tetrafluoride ThF_4_ has a well known monoclinic structure, in space group *C*2/*c* (Benner & Müller, 1990 ▸; Kern *et al.*, 1994 ▸), with two different thorium sites, both eightfold-coordinated, and with rather close Th—F bond distances. Because it is not possible to distinguish differences between coordination shells below ∼0.1 Å in EXAFS, the fitting was performed considering only one Th–F coordination shell, and one Th–Th first coordination shell. The first peak at ∼1.8 Å (Fig. 6 ▸) corresponds to the eight fluorine atoms surrounding the thorium, and the peak at ∼4.4 Å to the thorium first neighbour. The fitted bond distances are listed in Table 1 ▸, and compared with the distances obtained by Rietveld refinement (see Appendix *B* [App appb] for the refined lattice parameters and atomic positions) of neutron diffraction data^1^ (Fig. 7 ▸) collected on the same sample batch at the beamline PEARL at the Hoger Onderwijs Reactor at TU Delft (van Eijck *et al.*, 2016 ▸). The agreement is very good.

The distance resolution Δ*R*, *i.e.* the ability to differentiate neighbouring atom shells in the EXAFS spectrum, is given by Δ*R* = Π/2Δ*k* where Δ*k* is the *k*-range of the spectra. Δ*R* is equal to 0.17 Å for the present *k*-range.

### 4.2. Mapping of the concentration profile   

**Figure 8:**
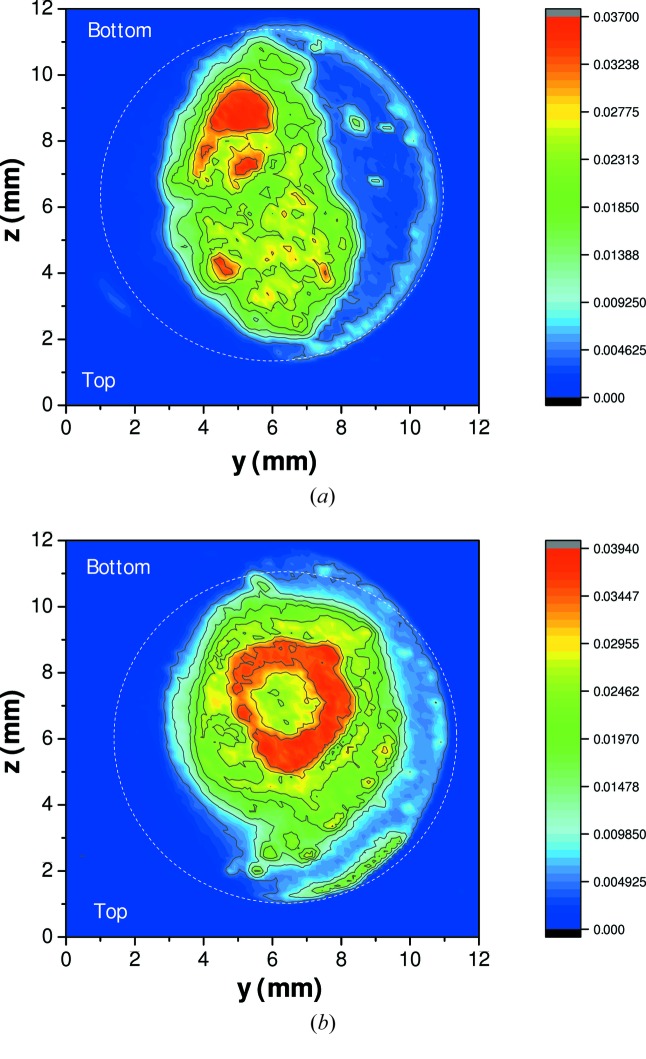
Mapping of the thorium concentration profile after melting of the (*a*) (NaF:ThF_4_) = (0.67:0.33) and (*b*) (NaF:ThF_4_) = (0.5:0.5) salt mixtures. The thorium fluorescence signal was collected at 17 keV while scanning the sample surface along the *y* and *z* axes. The colour scale corresponds to the fluorescence signal normalized to the intensity of the incident beam.

Next, the suitability of the encapsulation technique used in this work to contain the thorium salt was verified with a mapping of the thorium concentration profile. The end-members (LiF, NaF, ThF_4_) were thoroughly mixed during sample preparation to ensure a homogeneous distribution of material in the entire volume inside the reservoir of the BN cell. However, we wanted to check that the homogeneity could also be maintained upon melting. To this end, a couple of sample cells were recovered after the EXAFS measurements at high temperatures, and the thorium fluorescence signal was collected at an energy of *E* = 17.0 keV while scanning the sample along the *y* (lateral) and *z* (vertical) positions in the X-ray beam (the *x* axis being along the beam), over the entire sample surface. The resulting thorium concentration maps for the (NaF:ThF_4_) = (0.67:0.33) and (NaF:ThF_4_) = (0.5:0.5) salt mixtures are shown in Figs. 8(*a*) and 8(*b*) ▸. The homogeneous distribution is maintained upon melting over several hours, except for a few concentrated regions, but these remain at a sufficient distance from the rim of the cell to prevent any risk of a leak. Gamma spectroscopy measurements performed on the molten sample cells after collection of the EXAFS data also confirmed the absence of contamination on the outside rim of the BN containment.

### 4.3. EXAFS measurements on molten LiF–ThF_4_ and NaF–ThF_4_ salt mixtures   

**Figure 9:**
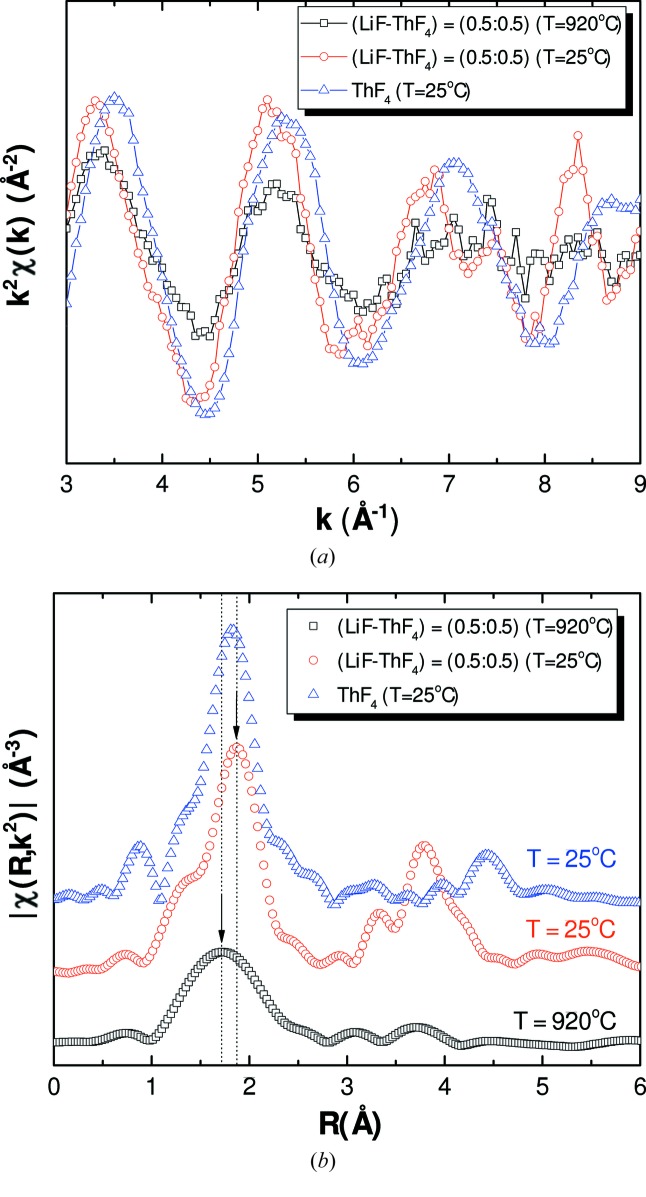
(*a*) Experimental *k* ^2^χ(*k*) spectra and (*b*) Fourier transform modulus of the (LiF:ThF_4_) = (0.5:0.5) sample measured at room temperature and *T* = 920°C. The reported data were collected in fluorescence mode. Comparison with the spectrum of ThF_4_ at room temperature, measured in transmission.

Using our dedicated experimental set-up, the EXAFS spectra of the (LiF:ThF_4_) and (NaF:ThF_4_) salt mixtures were recorded at high temperature in the molten state, and at room temperature after cooling and solidification. Figs. 9(*a*) and 9(*b*) ▸ show the experimental *k* ^2^-weighted EXAFS spectra and FT of the (LiF:ThF_4_) = (0.5:0.5) sample collected at *T* = 920°C and at room temperature after cooling. The EXAFS signal appears slightly shifted towards higher wavenumbers *k* in the molten state, which corresponds in the FT data to a first coordination shell Th–F at lower radial distances. One can also notice a characteristic decrease in amplitude of the EXAFS signal and of the first coordination shell in the FT, which is caused by the disorder in the liquid and the occurrence of anharmonic oscillations (Pauvert *et al.*, 2010 ▸; Bessada *et al.*, 2017 ▸). Moreover, one can clearly observe two coordination shells at room temperature after melting, against one single coordination shell in the liquid. These features distinctly confirm the liquid state of the sample during the high-temperature measurements. It is also worth pointing out the differing shapes of the signals of ThF_4_ at room temperature and of the re-solidified material, which corresponds to the LiThF_5_ ternary compound with tetragonal structure in space group *I*4_1_/*a* (Grzechnik *et al.*, 2013 ▸). In the latter spectrum, an intense second peak appears around ∼3.8 Å, which is absent in ThF_4_. This comparison gives further proof that the end-members have indeed reacted together in the liquid to form the ternary intermediate compound upon cooling.

The LiF–ThF_4_ and NaF–ThF_4_ fluoride salts are ionic liquids that form a dissociated ionic melt with [ThF_6_]^2−^, [ThF_7_]^3−^, [ThF_8_]^4−^, [ThF_9_]^5−^, [ThF_10_]^5−^ anionic complexes in varying proportions (Numakura *et al.*, 2014 ▸, 2016 ▸; Bessada *et al.*, 2017 ▸; Dewan *et al.*, 2013 ▸; Liu *et al.*, 2014 ▸; Dai *et al.*, 2015 ▸), [ThF_8_]^4−^ being the predominant complex for the ThF_4_ end-member composition (Numakura *et al.*, 2016 ▸). The EXAFS signal is the sum of all contributions corresponding to these anionic complexes, and the average coordination number (CN) varies with composition. Past studies have reported an average CN of about 7 for both systems in the 10–50% ThF_4_ composition range when fitting the EXAFS data to the standard EXAFS equation including *C*_3_ and *C*_4_ cumulants to account for the anharmonic oscillations at high temperature (Numakura *et al.*, 2014 ▸). Based on MD simulations of the salts, the same authors have reported an average CN of around 8, however. The traditional approach applied to solids is in fact not well adapted for highly disordered systems such as in the present case (Filliponi, 2001 ▸; Okamoto, 2004 ▸; Bessada *et al.*, 2017 ▸) due to a high correlation between the fitting parameters, and the use of MD to simulate the EXAFS data is to be preferred. The MD studies (Dewan *et al.*, 2013 ▸; Liu *et al.*, 2014 ▸; Dai *et al.*, 2015 ▸) on the LiF–ThF_4_, pure ThF_4_ and ThF_4_–LiF–BeF_2_ systems report the coexistence of [ThF_7_]^3−^, [ThF_8_]^4−^ and [ThF_9_]^5−^ complexes in the melt, the proportion of [ThF_6_]^2−^ and [ThF_10_]^5−^ being very minor, and the eightfold-coordinated complex [ThF_8_]^4−^ being the predominant one.

**Figure 10:**
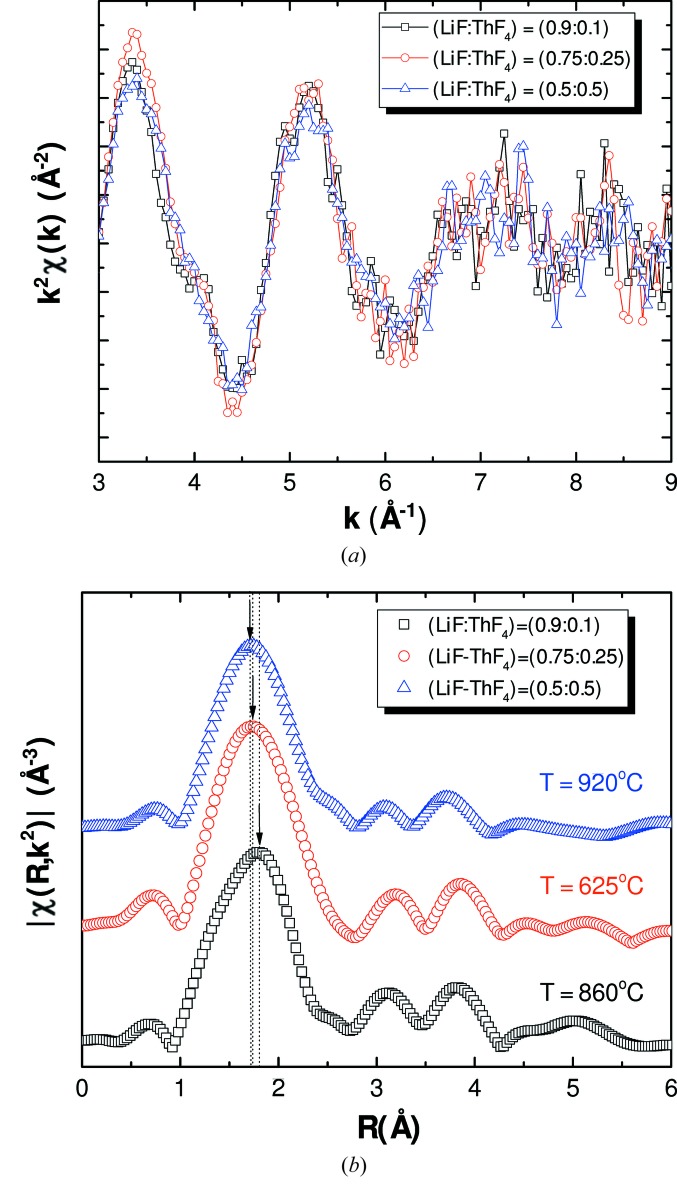
(*a*) Experimental *k* ^2^χ(*k*) spectra and (*b*) Fourier transform modulus of the LiF–ThF_4_ series measured at high temperature in fluorescence.

As can be seen in the EXAFS data measured in this work (Figs. 10 ▸ and 11 ▸), the first coordination shell is shifted in both systems to slightly lower radial distances with increasing ThF_4_ concentration, indicating a contraction of the average first Th—F distance. This feature can qualitatively be related to the polarizing effect of the Li^+^ and Na^+^ cations. The more counter-cations are present in solution, the more the F^−^ anions are pulled away from the Th^4+^ coordinating centre, resulting in a longer average Th—F distance.

**Figure 12:**
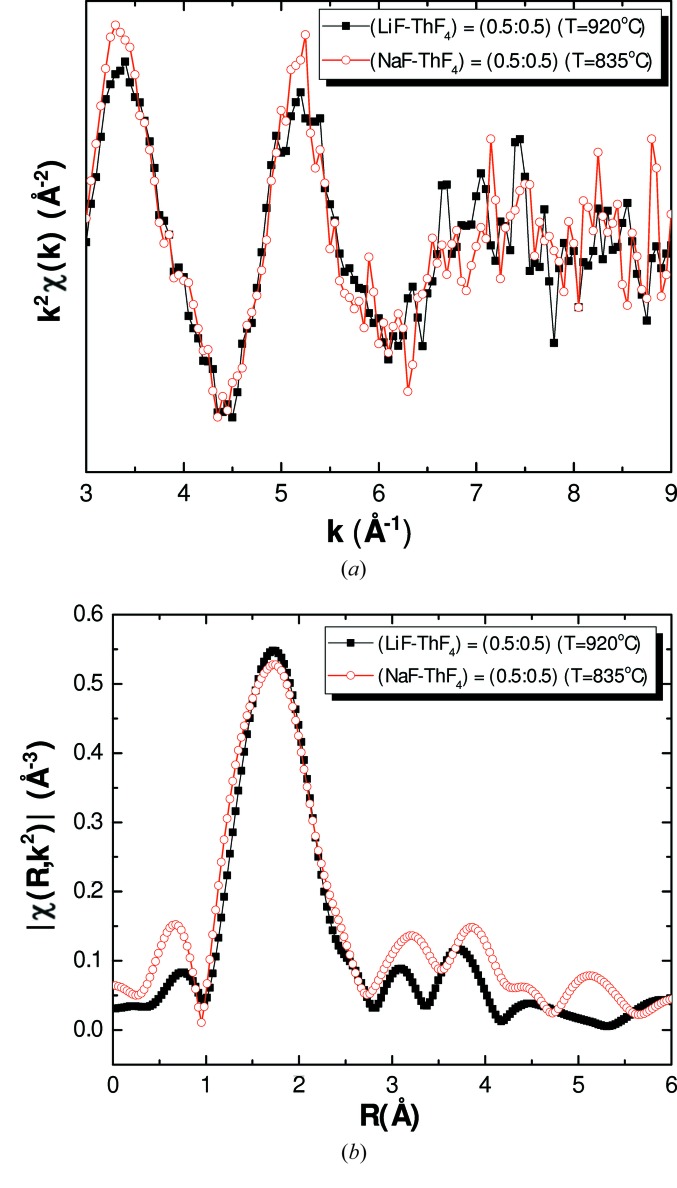
(*a*) Experimental *k* ^2^χ(*k*) spectra and (*b*) Fourier transform modulus of the (LiF:ThF_4_) = (0.5:0.5) and (NaF:ThF_4_) = (0.5:0.5) samples measured in fluorescence.

Finally, the comparison of the (LiF:ThF_4_) = (0.5:0.5) and (NaF:ThF_4_) = (0.5:0.5) salt mixtures reveals very similar EXAFS and FT spectra (Fig. 12 ▸). One would expect a first coordination shell slightly shifted to lower radial distances for the sodium-containing salt. The larger ionic radius of Na^+^ (0.99 Å) compared with Li^+^ (0.59 Å) means that the Li^+^ cations come closer to the fluoride anions surrounding Th^4+^, leading to a larger exchange rate, as reported by Numakura *et al.* (2014 ▸). As a consequence, the local structure around Th^4+^ is more stable in the NaF–ThF_4_ mixture (Numakura *et al.*, 2014 ▸). This feature is not really evident from the present experimental results, but could become more obvious when measuring the larger alkali metals (Rb, Cs). We should point out that these two measurements were carried out with an 85°C temperature difference, which will have an effect on the chemical speciation. However, we expect this effect to be very limited given the results obtained by Dewan *et al.* (2013 ▸) with MD simulations on the (LiF:ThF_4_) = (0.78:0.22) mixture in the temperature range 577–1000°C.

It is interesting to compare the behaviour of the *M*F–ThF_4_ mixtures with that of the *M*F–ZrF_4_ systems (*M* = Li, Na, K) (Pauvert *et al.*, 2010 ▸, 2011 ▸). Pauvert *et al.* reported the formation of [ZrF_6_]^2−^, [ZrF_7_]^3−^ and [ZrF_8_]^4−^ complexes based on coupled EXAFS measurements and MD simulations, with a progressive stabilization of the sevenfold- and sixfold-coordinated complexes with increasing size of the alkali cation along the series Li–Na–K (Pauvert *et al.*, 2010 ▸, 2011 ▸), and an increase in the distance between octahedral units. A similar behaviour can be expected in the *M*F–ThF_4_ series (*M* = Li, Na, K, Cs, Rb). The recent measurements of Bessada *et al.* (2017 ▸) on a (KF:ThF_4_) = (0.75:0.25) salt mixture at 900°C coupled with MD simulations have yielded an average coordination number of 7.03 with the following distribution of complexes: [ThF_6_]^2−^ (14.5%), [ThF_7_]^3−^ (68.7%) and [ThF_8_]^4−^ (16.8%). It would be highly interesting to perform measurements in the CsF–ThF_4_ and RbF–ThF_4_ systems to confirm whether complexes with a lower coordination number are indeed favoured.

### 4.4. Fitting and MD simulations of high-temperature EXAFS data for (LiF:ThF_4_) = (0.5:0.5)   

**Figure 13:**
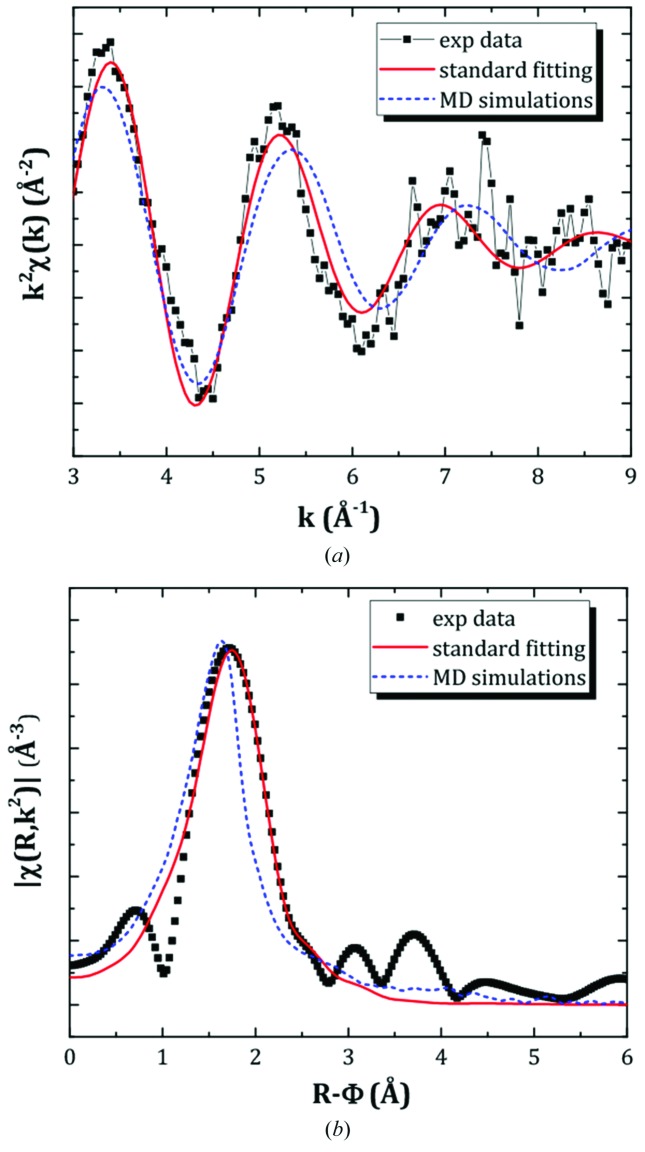
(*a*) Experimental and fitted *k* ^2^χ(*k*) and (*b*) Fourier transform modulus of the sample of composition (LiF:ThF_4_) = (0.5:0.5) at 920°C. The goodness of fit is *R*_f_ = 0.018 using the standard EXAFS equation. The data are also compared with the MD simulated spectra.

The experimental data for the (LiF:ThF_4_) = (0.5:0.5) composition were fitted using the standard EXAFS equation, considering a single first coordination shell. The experimental and fitted *k* ^2^-weighted EXAFS spectrum and its FT are shown in Figs. 13(*a*) and 13(*b*) ▸. The fitted parameters were found to be: *R* = 2.33 (1) Å (interactomic average Th—F distance), CN = 7.3 (3) (average coordination number), σ^2^ = 0.019 (1) (Debye–Waller factor) and Δ*E*_0_ = 3.29 eV (energy shift). The rather large Debye–Waller factor results from the thermal and structural disorder in the liquid. The inclusion of a *C*_3_ cumulant accounting for the disorder was considered during the fitting procedure. But no real improvement was obtained, as the reduction of the goodness-of-fit *R*_f_ factor was less than 0.3%, while the induced modifications in the metric data (CN, *R*, σ^2^) were within initial uncertainties. Furthermore, given that the uncertainty on *C*_3_ was three times the value itself, the presence of an asymmetry was discarded in our data. Further experiments should allow confirmation or not of this observation. As seen in Figs. 13(*a*) and 13(*b*) ▸, the agreement between experimental data and calculated curve is very good. The fitted bond distance is, moreover, as should be expected, slightly higher than that reported by Bessada *et al.* for a sample of composition (KF:ThF_4_) = (0.75:0.25) measured at 900°C (Bessada *et al.*, 2017 ▸). The authors reported *R* = 2.237 (22) Å and *R* = 2.29 Å based on fitting with the standard EXAFS equation and MD simulations, respectively. The fitted average coordination number is of the same order of magnitude as in the work of Bessada *et al.* [CN ≃ 7.1 (10.4) based on standard fitting, CN ≃ 7.03 based on MD simulations].

Finally, the same experimental data were compared with the EXAFS spectrum generated via MD simulations, a method which has been used in the literature for highly disordered fluoride salts in the liquid state (Pauvert *et al.*, 2011 ▸; Bessada *et al.*, 2017 ▸) as mentioned before. Once the NVT production run was computed, the series of extracted MD trajectories were used as input for the *FEFF8.40* code (Ankudinov *et al.*, 1998 ▸) in order to compute the EXAFS signal, which was then directly compared with the experimental data. The generated signal was moreover constituted of the accumulation of 25000 atomic configurations so as to reproduce the effect of the Debye–Waller factor and the anharmonic vibrations. Figs. 13(*a*) and 13(*b*) ▸ compare the experimental and the calculated *k* ^2^χ(*k*) curve and FT modulus for (LiF:ThF_4_) = (0.5:0.5) at 920°C. The agreement is reasonably good on the third oscillation of the *k* ^2^χ(*k*) signal but rather poor on the first and second oscillations. The average Th—F distance obtained from the MD simulations, *i.e.* ∼2.23 Å, is about 0.1 Å lower than that derived by the standard fitting procedure, explaining the discrepancy with the experimental data. This result suggests that the MD potentials need to be slightly adjusted, which will be done in future work. It also stresses the usefulness of such experimental work as a benchmark for MD computations and to help improve the MD models. The MD potentials, once they are well defined, can be used to determine a number of thermochemical and thermophysical properties (heat capacity, density, viscosity, electrical conductivity, *etc.*) in a wide range of compositions and temperatures, which might be very challenging and/or costly to measure experimentally (Dewan *et al.*, 2013 ▸; Liu *et al.*, 2014 ▸).

**Figure 14:**
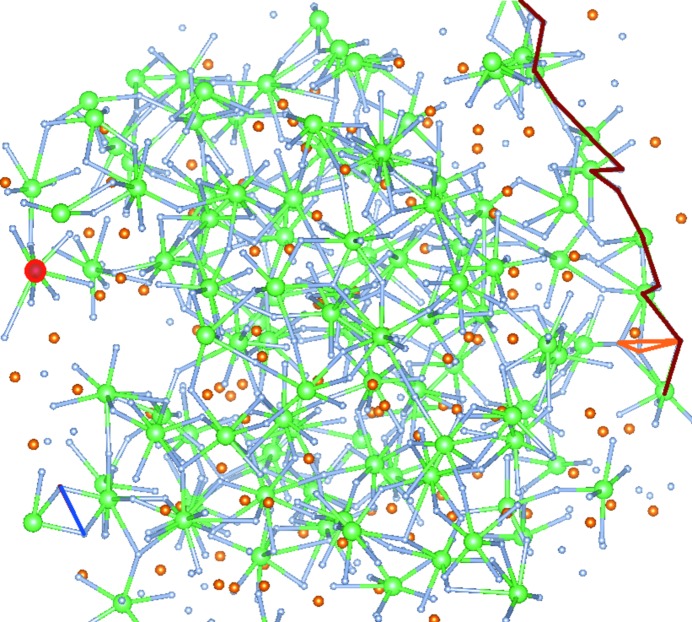
MD snapshot of the atomic configurations calculated for the (LiF:ThF_4_) = (0.5:0.5) composition at *T* = 920°C. Orange: Li^+^ ions; green: Th^4+^ ions; grey: F^−^ ions. Li^+^–Th^4+^ pairs at a distance less than the minimum of the radial distribution function (3.2 Å) are shown as bonds and considered to belong to the coordination sphere of the thorium. Red: seven-coordinated Th^4+^; blue: edge-sharing polyhedra; orange: face-sharing polyhedra; brown: 7 Th-long chain.

**Figure 15:**
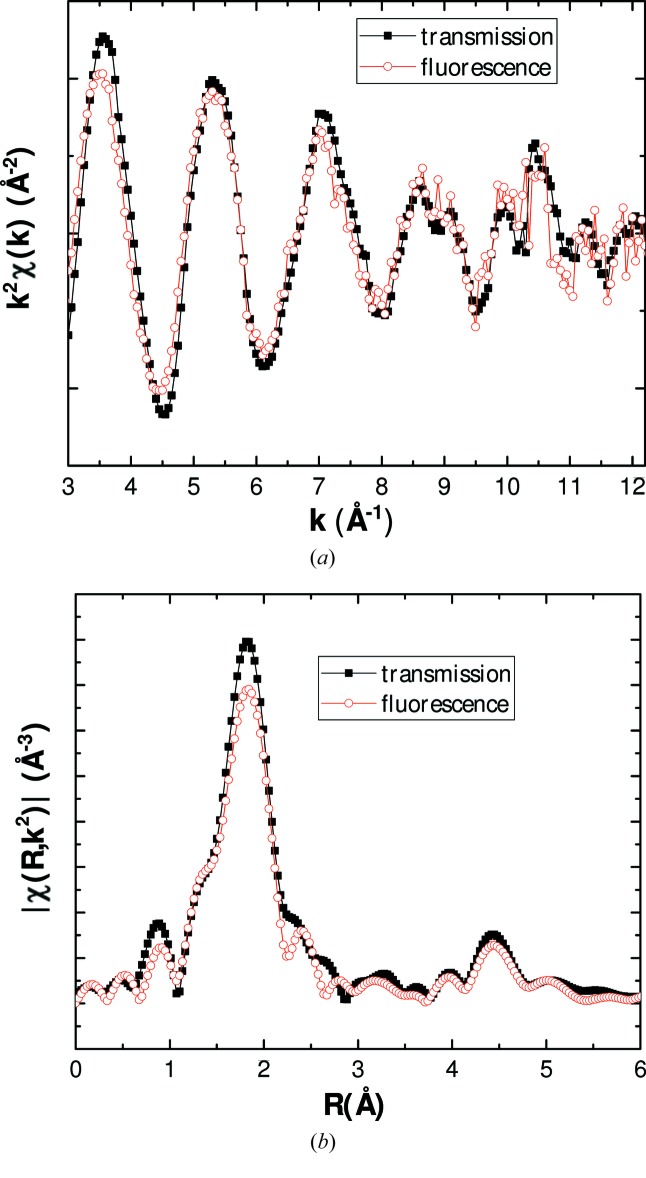
(*a*) Experimental *k* ^2^χ(*k*) spectra and (*b*) Fourier transform modulus of ThF_4_ measured at room temperature in transmission and fluorescence. Note that the fluorescence spectrum was not corrected for self-absorption effects.

As Pauvert *et al.* (2011 ▸) point out, the benefit of using MD to interpret EXAFS data of liquid samples is that a complete description of the short-range structure is available: coordination numbers can be assigned with a simple geometric criterion. The radial distribution function (RDF) of the anions around the cation of interest is calculated, and the first local minimum is assigned to be the bond cut-off distance. Any anion closer to the cation than the minimum of the RDF is said to belong to the first coordination shell of the cation. In the case of (LiF:ThF_4_ = 0.5:0.5), the minimum of the RDF is calculated close to 3.2 Å, and the following distribution of complexes centred around Th^4+^ is obtained: [ThF_6_^2−^] = 3.3%, [ThF_7_^3−^] = 28.2%, [ThF_8_^4−^] = 49.5%, [ThF_9_^5−^] = 17.5% and [ThF_10_^6−^] = 1.5%. That is, the sevenfold-, eightfold- and ninefold-coordinated Th^4+^ ions predominate, and the average coordination number is CN = 7.9. These complexes are not all isolated from each other but actually form networks: only 5.4% of them are not connected to another cluster, while 51.5% share one bridging fluorine, 35.4% share two fluorines (an edge of their respective polyhedra) and 7.7% share three fluorines (a face of their respective polyhedra) (Fig. 14 ▸). Amongst the complexes which form a chain, 0.8% are linked to only one other complex, and those linked to 2, 3, 4, 5 and 6 complexes represent 4.6%, 22.3%, 36.7%, 27.8% and 7.8%, respectively (Fig. 14 ▸). Such a network structure is not surprising since the small ionic radius of Li^+^ ensures it can be incorporated within the network, as has been shown for other network-forming liquids (Wilson & Madden, 1994 ▸). The network-disrupting abilities of larger alkali ions such as Rb and Cs, as well as their effect on coordination and average distance, will be explored in future studies using our newly developed experimental set-up.

## 5. Conclusions   

A dedicated experimental set-up for the EXAFS measurements of molten fluoride salt materials has been developed in this work and successfully tested for several hours at high temperatures. It is the first time that such a design is reported that allows simultaneous measurements in transmission and fluorescence, offering a good flexibility with respect to sample composition, from very diluted to very concentrated materials. Moreover, the developed air-tight sample containment cell, made of boron nitride, allowing measurements on pure materials, has shown good results with respect to signal quality, and has proved reliable against leakage and possible contamination as shown by the post-analysis characterizations (mapping of the thorium concentration profile and gamma spectroscopy measurements). The fitted EXAFS data for thorium tetrafluoride at room temperature were found to be in good agreement with the refined bond distances obtained with a Rietveld refinement of neutron diffraction data. The EXAFS data collected on selected molten salt mixtures in the LiF–ThF_4_ and NaF–ThF_4_ systems have confirmed trends reported in the literature: (i) shift of the EXAFS signal to higher wavenumber *k* in the molten state due to contraction of the Th—F distances in the first coordination shell; (ii) decrease in signal amplitude due to increased disorder and anharmonic effects; (iii) shift in the FT signal of the first coordination shell to lower distances with increasing ThF_4_ content.

Finally, the experimental data for the (LiF:ThF_4_) = (0.5:0.5) composition were compared with the EXAFS signal generated from MD simulations, which showed some differences and which will be explored further. This result shows that such experimental studies are highly relevant for validation and further improvement of simulation tools.

This set-up will be used in future investigations to determine coordination numbers and bond distances of the molecular complexes formed in various fluoride salt mixtures, which is a very powerful tool for the investigation and optimization of the physico-chemical properties of the fuel of molten salt reactors.

## Supplementary Material

Crystal structure: contains datablock(s) I. DOI: 10.1107/S160057751801648X/hf5371sup1.cif

CCDC reference: 1879921

## Figures and Tables

**Figure 5:**
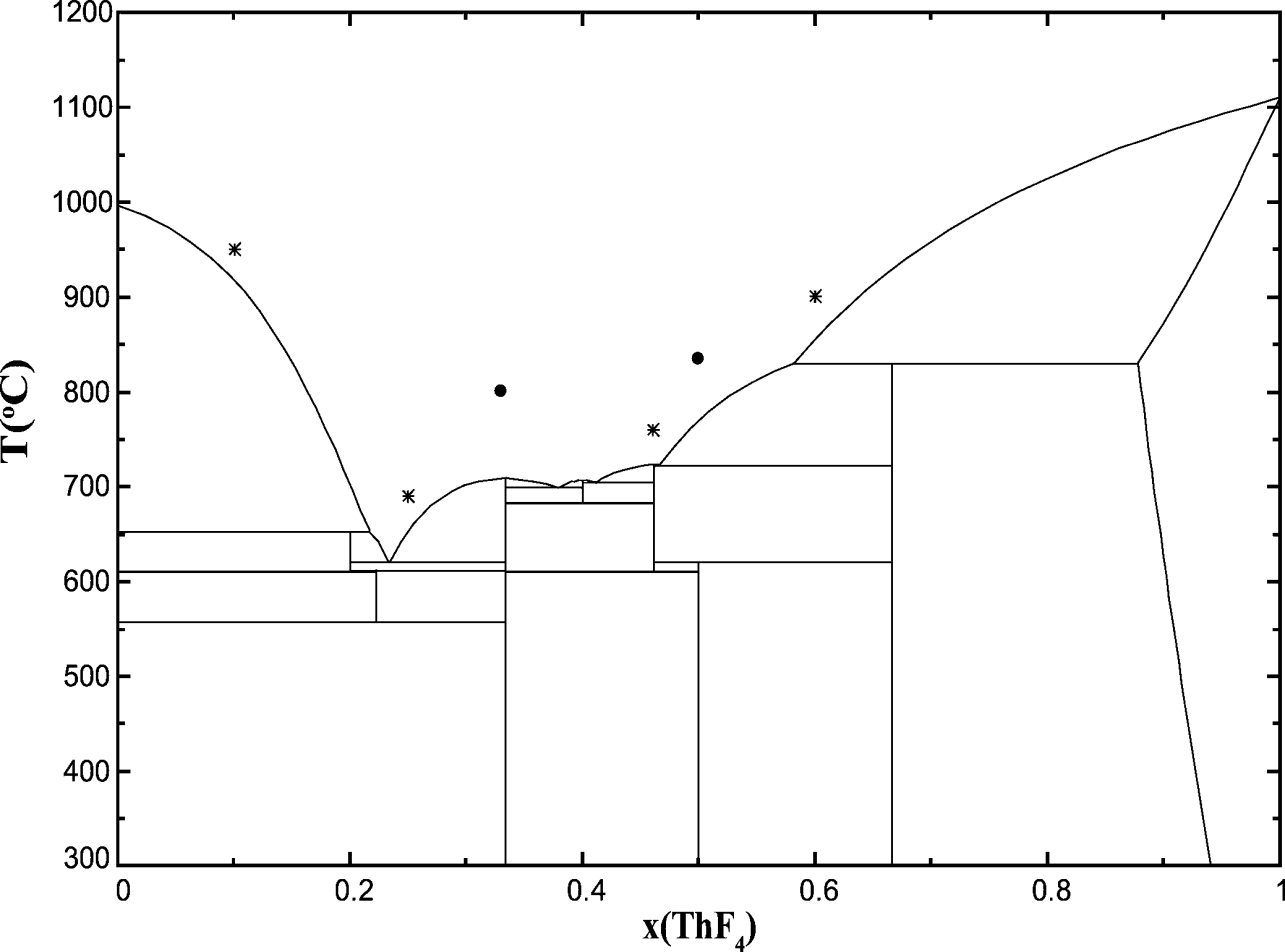
NaF–ThF_4_ phase diagram as optimized in the work of Capelli *et al.* (2014 ▸). Filled circles: compositions measured in this work; stars: compositions reported by Numakura *et al.* (2014 ▸): note that the EXAFS data are not shown in this reference, and the temperature of the measurement is not specified.

**Figure 11:**
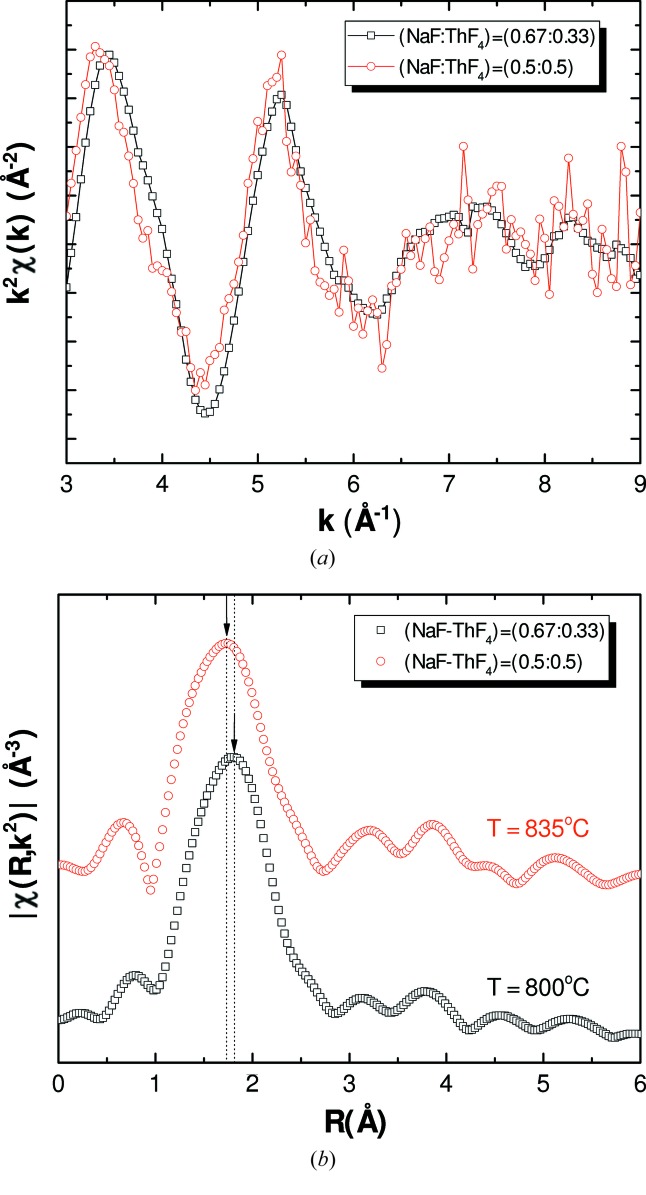
(*a*) Experimental *k* ^2^χ(*k*) spectra and (*b*) Fourier transform modulus of the NaF–ThF_4_ series measured at high temperature in fluorescence for (NaF:ThF_4_) = (0.5:0.5) and absorption for (NaF:ThF_4_) = (0.67:0.33).

**Table 1:** Selected bond lengths *R*(Å) for ThF_4_ derived from the present study using neutron diffraction (goodness of fit of the Rietveld refinement: χ^2^ = 7.84, *R*_wp_ = 22.4, *R*_exp_ = 8.00; background: linear interpolation between operator-selected points in the pattern with refinable heights) and EXAFS spectroscopy (Δ*E*_0_ = −0.62 eV, goodness of fit *R*_f_ = 0.025) Standard deviations are given in parentheses. *N* is the number of atoms in each shell. σ^2^ is the Debye–Waller factor.

Bond	*N*	*R* (Å)	σ^2^ (Å^2^)
Neutron data			
Th1—F7	1	2.269 (11)	
Th1—F6	1	2.27 (3)	
Th1—F4	1	2.286 (10)	
Th1—F3	1	2.31 (3)	
Th1—F5	1	2.317 (13)	
Th1—F2	1	2.332 (19)	
Th1—F7	1	2.344 (13)	
Th1—F1	1	2.396 (12)	
Th2—F2	2	2.27 (3)	
Th2—F3	2	2.295 (16)	
Th2—F5	2	2.359 (18)	
Th2—F6	2	2.41 (2)	
Th1—Th1	2	4.554 (9)	
Th2—Th1	2	4.635 (14)	

Averaged neutron data			
Th—F	8	2.324 (19)	
Th—Th	4	4.594 (12)	

EXAFS data			
Th—F	8	2.30 (1)	0.0060 (5)
Th—Th	2	4.61 (1)	0.0003 (10)

**Table 2:** Refined atomic positions in ThF_4_ derived from the neutron refinement The refined lattice parameters are *a* = 13.025 (9) Å, *b* = 11.005 (7) Å, *c* = 8.528 (5) Å, β = 126.27 (4)°. *R*_wp_ = 22.4, *R*_exp_ = 8.00, χ^2^ = 7.84. Background: linear interpolation between operator-selected points in the pattern with refinable heights.

Atom	Oxidation state	Wyckoff	*x*	*y*	*z*	*B*_0_ (Å^2^)
Th1	+4	8f	0.20715 (94)	0.42744 (81)	0.33207 (140)	0.9 (1)
Th2	+4	4e	0	0.78390 (120)	0.25	0.8 (2)
F1	−1	8f	0.5	0.88551 (240)	0.25	1.4 (5)
F2	−1	8f	0.11443 (141)	0.61850 (145)	0.27891 (229)	0.7 (2)
F3	−1	8f	0.12990 (143)	0.84128 (139)	0.16364 (205)	0.7 (2)
F4	−1	8f	0.25	0.75	0	1.0 (4)
F5	−1	8f	0.87877 (133)	0.94741 (126)	0.04207 (205)	0.5 (2)
F6	−1	4e	0.89517 (153)	0.69835 (170)	0.93031 (228)	0.9 (3)
F7	−1	4c	0.21044 (153)	0.53249 (135)	0.09633 (225)	0.6 (2)

## Footnotes

1 The sample for the neutron diffraction (∼1 g) was encapsulated in a vanadium container hermetically closed with a rubber O-ring. The data were collected at room temperature, at a fixed wavelength (λ = 1.667 Å) for 3 h over the range 11° ≤ 2θ ≤ 60°. Structural analysis was performed by the Rietveld method with the *Fullprof2k* suite (Rodríguez-Carvajal, 1993 ▸).

## Appendix A ThF_4_ room-temperature EXAFS data

Fig. 15 ▸ shows the experimental *k* ^2^(*k*) spectra and FT modulus of ThF_4_ measured at room temperature in transmission and fluorescence.

## Appendix B Structural refinement of ThF_4_

Table 2 ▸ shows the structural refinement data of ThF_4_ based on room-temperature neutron diffraction data collected at the PEARL beamline, TU Delft, The Netherlands.
